# Inflammatory Bowel Disease in Australasian Children and Adolescents

**DOI:** 10.1155/2014/703890

**Published:** 2014-03-30

**Authors:** A. S. Day, D. A. Lemberg, R. B. Gearry

**Affiliations:** ^1^Department of Paediatrics, University of Otago (Christchurch), Riccarton Avenue, Christchurch 8140, New Zealand; ^2^Department of Paediatric Gastroenterology, Sydney Children's Hospital, Randwick, Sydney, NSW 2031, Australia; ^3^Department of Medicine, University of Otago (Christchurch), Riccarton Avenue, Christchurch 8140, New Zealand

## Abstract

Many reports indicate increasing rates of inflammatory bowel disease, with data also showing changing patterns of this chronic disease in children and adolescents. This review focuses upon the available data of the epidemiology of inflammatory bowel disease in children and adolescents in Australia and New Zealand (collectively termed Australasia). Recent data show high incidence of IBD (especially Crohn disease) in this area and indicate rising rates of IBD in children and adolescents.

## 1. Introduction

There are numerous reports documenting increasing rates of the inflammatory bowel diseases (IBD), Crohn disease (CD), and ulcerative colitis (UC), over recent years [1]. Whilst initial publications focused on the incidence and prevalence of CD and UC in western populations, a number of reports have also described increasing rates in other parts of the world, especially Asian countries [2].

Furthermore, paediatric reports have noted increasing rates of IBD in children and adolescents [3–5]. Reports from various countries especially show higher rates of CD, and some of these publications also show onset in younger children.

Recent data from New Zealand (NZ) and Australia (collectively known as Australasia) indicate high incidence of IBD, especially CD [6, 7]. Several reports also indicate increasing diagnoses in children and adolescents [8, 9]. This review focuses on the patterns of CD and UC in Australasian children and adolescents whilst illustrating some key aspects of IBD in this region of the world.

## 2. Patterns of IBD in New Zealand and Australia

Prior to 2006, the only reports of IBD in Australia [10, 11] or NZ [12–14] were small hospital-based or tertiary case series. Several of these reports focused on series of patients, especially describing surgical experiences [10, 11, 13]. Overall, these reports suggested low incidence and prevalence of IBD [12, 14]. More recently there have been comprehensive and prospective population-based studies that have more clearly illustrated key aspects of IBD in Australasia.

The first of these studies was a population-based study of IBD in the Canterbury province in the South Island of NZ [6]. Using overlapping methodologies, including capture/recapture, the investigators established a population of 1420 individuals, which was estimated to represent more than 91% of those diagnosed with IBD within this geographical region. This population included those with existing disease and an incidence cohort (*n* = 116) collected prospectively over the calendar year of 2004. This cohort established an incidence of 16.5 per 100,000 for CD and incidence of 7.6 per 100,000 for UC (Table 1). The point prevalence (as at January 1st, 2005) was calculated for IBD, CD, and UC to be 308.3, 155.2, and 145/100,000, respectively. These incidence and prevalence data were amongst the highest rates for CD at that time.

These high rates of CD seen in Canterbury are supported in a further population-based study from the Nelson region in the north of the South Island of NZ (unpublished data, RB Gearry). The region of Nelson is a distinct geographical area that features a stable population served by one secondary level hospital. A population-based assessment of individuals diagnosed with IBD in the Nelson area delineated 224 individuals with CD and 160 with UC. The overall prevalence of IBD was calculated to be 389/100,000 population. The incidence of CD was noted to be rising over the last decade, with incidence for the 2012 year calculated as 15.2/100,000: one of the highest rates reported to date (Table 1). Ongoing prospective assessment of incidence rates in this well-defined area will provide valuable information on the epidemiology of IBD in NZ.

A further population-based study focused upon a well-defined population in the Geelong area in the Australian state of Victoria [7]. This study was conducted over a one-year period from April 2007 within a specific geographic region comprising a total population of 259,015 people. Seventy-six new diagnoses of IBD were made over this period: 45 CD, 29 UC, and two IBDU. The overall incidences of CD and UC were calculated to be 17.4 and 11.2 per 100,000, respectively (Table 1). The peak age at diagnosis was in the 20–24-year group: just fourteen (21%) of the total group were less than 22 years of age at diagnosis.

These prospective population-based studies have together demonstrated high rates of IBD in Australasia. They have particularly indicated a high incidence of CD in both countries. Although these data likely represent the broader populations in the two countries, comparative studies in other locations have not yet been reported.

In addition, a further study arising from the Canterbury cohort study examined environmental factors contributing to the development of IBD [15].

This study incorporated a case-control design, with the cases comprising 638 individuals with CD and 653 with UC from the wider cohort. Six-hundred age and sex-matched controls without IBD were included for comparison. All subjects were asked to complete a questionnaire informing their current or previous exposure to various environmental risk factors. Key factors associated with IBD were higher socioeconomic status, European ethnicity, family history of IBD, and smoking history at diagnosis. Family history was the strongest factor (odds ratio of 3.1 for CD and 2.5 for UC). Living in an urban location was linked with risk of CD, whilst those who had moved to the region from another country were more likely to have developed UC. In contrast, breast feeding in infancy and having a vegetable garden in childhood were both protective factors against the development of IBD. This population-based case-control study confirmed the findings of prior work in regard to the protective benefit of breast-feeding [16] and has emphasised the importance of early life events (e.g., rural living) that may modify or modulate the development of the intestinal microflora and thereby influence the risk of developing IBD.

A further recent study has also looked at environmental risk factors for IBD: this report focused upon immigrants moving to Australia from the Middle East [17]. The study included 84 adults of Middle Eastern ethnicity with IBD seen and assessed in Sydney, Australia, with comparison to European cases and controls, and with Middle Eastern controls in Australia and in Lebanon. Family history of IBD was a key risk factor. Breast-feeding, pet ownership, rural residence, and contact with farm animals were shown to be additional factors modifying the risk of IBD. Within families that had migrated to Australia from the Middle East, diagnosis of IBD was seen earlier in the second generation than in the first generation. A number of the risk factors for IBD (such as rural dwelling) were also more prominent in the second generation of migrants. As in the NZ cohort, many of the key factors reflected early life events (e.g., breast feeding) and factors influencing the development of the intestinal microflora.

## 3. IBD in the Indigenous Peoples of Australasia

Although Australasia is predominantly populated by individuals of European ethnicity, both Australia and NZ have indigenous populations: Aborigines and Maori, respectively.

The total group of patients assembled in the Canterbury population-based cohort were predominantly (97.5%) of European extraction, with only 14 (0.98%) being of Maori ethnicity [6]. Eight (1.1%) of the 715 patients with CD were Maori. At this time, Maori people represented 7.3% of the Canterbury population and approximately 15% of the total NZ population. Earlier hospital-based studies in NZ have also indicated that IBD is very uncommon in Maori people [12–14]. These studies have also indicated that IBD is uncommon in other Polynesian peoples resident in NZ, such as those from the islands of Samoa and Tonga.

As the data incorporated in the Geelong study did not include ethnicity data, rates of IBD in Aborigines were not available to be ascertained in that report [7]. However, data obtained from the Australian Paediatric and Adolescent IBD Database, a prospective registry commenced in 1996, suggests very low rates of IBD in children of Aboriginal extraction compared to non-Aboriginal Australian children [18]. Only 13 (0.57%) of 2300 children included in the database came from families with one or two parents of Aboriginal descent. Based upon the 2006 Australian census, Aborigines comprise around 5% of the overall Australian population. This census data was also used to estimate 2006 point prevalence figures: these suggested rates of 5.2 (per 100,000) for Aboriginal children and 47.9 (per 100,000) for non-Aboriginal children. The census data also illustrated that the Aboriginal population has similar rates of urban and rural living compared to non-Aboriginal populations, suggesting that higher rural exposure does not explain the variation in IBD prevalence. Furthermore, Aboriginal populations appear to have adopted similar patterns of diet and lifestyle to Europeans, reflected in similar or higher rates of lifestyle-related disease, such as cardiovascular disease [19]. These observed differences could also be consequent to lower recognition (i.e., underreporting) of IBD in this population. This was not able to be explored further in this study, but given the significant and broad impacts of IBD upon patients, this would seem unlikely to explain the huge differences.

Interestingly, low rates of IBD are also observed in the indigenous peoples of Canada [20]. The reasons for lower rates in these three distinct indigenous populations (contrasting to higher rates in the predominantly European peoples in the three countries) are not fully characterised but could reflect a number of factors, including those relating to the pathogenesis of IBD, such as genetic differences or variations in enteric infections. Socioeconomic factors and access to healthcare could also be important variables.

In regard to important genes related to risk of IBD, one NZ study evaluated rates of CARD15 polymorphisms in 90 Maori and 201 European individuals [21]. Overall a low frequency of polymorphisms was observed in NZ Maori. A linear relationship was described between the presence of polymorphisms and increasing ancestry. This study indicated that Maori are much less likely to carry this risk gene and could contribute to lower rates of IBD. However, low rates of CARD15 polymorphism carriage are also seen in other ethnic groups, such as in Japanese individuals [21]. Furthermore, the rates of other risk genes have not been fully considered in the Maori population.

We have previously hypothesised that differences in gastric and enteric infections could also influence the development of IBD in Maori in NZ [22]. Maori have higher rates of* Helicobacter pylori* colonisation in childhood than Europeans in the same community [23]. Higher rates of this gastric infection have also been seen in Canadian First Nation peoples [24]. In contrast, rates of Campylobacter infection appear to be lower in NZ Maori populations than in respective Europeans [22]. Other studies have indicated low rates of* H. pylori* infection in individuals diagnosed with IBD, suggesting that this provides a protective environment against the development of this chronic inflammatory condition [25]. This could reflect that* H. pylori* colonisation provides a degree of protection against subsequent enteric infections that then trigger the onset of IBD. Further work is required to further understand these complex interactions.

## 4. Paediatric IBD in Australia

A series of reports from Australia have described patterns of IBD in Australian children. Increasing rates of CD and UC in recent years have clearly been demonstrated in several of these publications.

Schildkraut et al. [8] retrospectively evaluated the patterns of UC in children aged 16 years or less in the Australian state of Victoria over a 60-year period from January 1950. The authors determined that 342 children were diagnosed with UC over this time, with a sixty-fold increase in the incidence over the period, with this change being pronounced in the final ten years of observation. The incidence in 2009 was calculated to be 1.61 per 100,000 population.

The authors also delineated presenting features and disease phenotype in these children. Almost all of the children (93%) had been born in Australia and Indian or East Asian children comprised just 3% of the total group. Other details of ethnicity (such as Middle-Eastern background) were not provided. Diarrhoea and rectal bleeding were common symptoms at diagnosis, and children had a median of 16 weeks of symptoms before presentation. Of the 296 children where disease location was clearly defined at diagnosis, two thirds had pancolitis, with one quarter having left-sided disease and isolated proctitis seen in only 9%. Overall the children were lighter and shorter than the reference population (weight* z* score −0.21 and height* z* score −0.91).

Although not considered further in this publication, Schildkraut et al. [8] also identified 825 children diagnosed with CD and 143 with IBD unclassified (IBDU) in Victoria over this 60-year period. An earlier report had retrospectively reviewed children diagnosed with CD in the same area over a 31-year period (from 1971 to 2001) [9]. In this series of 351 children, the incidence of CD increased more than fifteenfold to 2.0 per 100,000 population. The presenting features, duration of symptoms prior to diagnosis, or anthropometric data were not included in this report. Ethnicity data were also not available. In terms of disease phenotype at diagnosis in the last decade of the study, 102 children (45%) were found to have upper gastrointestinal involvement, with 93% having colonic involvement and 57% having ileal involvement. Overall, 18 children were also noted to have perianal disease at diagnosis, whilst 21 had perioral involvement.

Together, these two reports from the same area of Australia show large increases in the numbers of children and adolescents diagnosed with IBD, with most of the changes observed in recent years.

A further study evaluated the phenotype and patterns of disease in a group of 86 children diagnosed with IBD at one tertiary hospital over a four-year period [26]. Sixty-one (71%) of this group were diagnosed with CD, 13 (21%) with UC, whilst 12 (20%) were labelled as IBDU. The children ranged from 0.6 years to 16.6 years (mean 9.8 years) at diagnosis and 55% were male. Forty-two of children with CD had diarrhoea, thirty-six had abdominal pain, and thirty-one (51%) had poor weight gains or weight loss before diagnosis.

Overall, 38 (44%) of the full group were found to have endoscopic and histological evidence of pancolitis [26]. Twenty-five of these 38 children were diagnosed with CD: in thirteen of this group the diagnosis of CD was based on upper gastrointestinal tract findings, including granulomatous gastritis or duodenitis. In contrast, nine children diagnosed with CD had no endoscopic or histological abnormalities on ileocolonoscopy—their diagnosis was based solely upon changes located in the upper gastrointestinal tract. This case series focused upon the presentation and location of disease, especially with regard to involvement in the upper gut: this study was not designed to delineate any changes over time.

One other Australian publication also focused on the patterns of upper gastrointestinal tract involvement in children diagnosed with CD [27]. This study showed upper gut involvement in 71% of 56 children diagnosed with CD. In 41% of these children, the findings detected in the upper gut were critical in making a diagnosis of CD.

A further Australian study has evaluated early life events in the risk of developing CD [28]. This study utilised a birth cohort born in Victoria between 1983 and 1998. Within this cohort, 278 children were diagnosed with CD by their 16th birthday. Perinatal factors linked with risk of CD included urban location, higher socioeconomic status, and delivery by caesarean section. In addition, the children born between 1992 and 1998 were 1.54 times more likely to have developed CD than the children born earlier. This difference was not related to changes in perinatal factors.

One study has focused on 42 Australian children diagnosed in the first six years of life (unpublished data, DA Lemberg). Twenty-four of this group were male and the median age at diagnosis was 3 years (range from 0.6 to 5.9 years). Two-thirds of the children (*n* = 28) were diagnosed with CD, whilst four were diagnosed with UC and ten labelled as IBDU at diagnosis. A first degree family history was seen in 8 children (19%) and most of the children (88%) had European background. Other ethnicities included Jewish, Indian, Australian Aboriginal, and Middle Eastern. These children predominantly presented with diarrhoea, rectal bleeding, and abdominal pain (present in 83%, 81%, and 52% of children, resp.) and symptoms were present for an average of 37.9 (±29.9) weeks.

Only one retrospective study to date has focused on rates of IBD in children within a specific population immigrating to Australia. The patterns of IBD in a group of 24 children of Middle-Eastern ethnicity were contrasted to age- and gender-matched children from non-Middle Eastern backgrounds within the IBD Clinic at a Sydney tertiary hospital [29]. Using 2006 population data, estimates of point incidence and point prevalence were 33.1 per 100,000 per year and 165.4 per 100,000, respectively, for the Middle-Eastern group. In contrast, the comparative rates in the control population were 4.3 per 100,000 and 28.7 per 100,000. Fourteen of the 24 Middle-East children had CD, seven had UC, and three had IBDU.

Some aspects of the phenotype and patterns of disease differed between the study and control groups [29]. At diagnosis the children of Middle-Eastern extraction had higher Pediatric Crohn Disease Activity Index (PCDAI) scores (37 ± 13 versus 27 ± 11, *P* = 0.033) and less colonic disease (*P* = 0.01) at diagnosis than the control group. The duration or patterns of symptoms before diagnosis did not differ. Growth data at diagnosis were similar in the two groups. The Middle-Eastern children had higher rates of thiopurine use after diagnosis (*P* = 0.002). Other outcomes (medical or surgical) did not differ between the two groups. Although this work included a relatively small group of patients, the patients were well characterised and were all seen and managed within one IBD Clinic. The role of early-life events and other environmental factors was not explored in this cohort.

## 5. Paediatric IBD in New Zealand

Within the NZ population-based cohort assembled by Gearry et al. [6] 126 patients were diagnosed prior to their 17th birthday. Eighty-five (67%) of this group were diagnosed with CD, whilst 40 (32%) had UC and one had IBDU. Half the patients were male. Other than two patients with Maori ancestry and one Asian child, all the other children were of European background.

Of the 83 children with CD who had adequate details of disease location, 27 had ileal disease, 32 colonic, and 24 ileocolonic location [30]. These children were more likely to have ileocolonic disease than adults diagnosed over the age of 40 years of age. Sixty-four children had inflammatory disease at diagnosis, with 9 having structuring disease and 11 having penetrating disease: penetrating disease at diagnosis was more common in children than in adults aged over 40 years. Over time, however, age at diagnosis was not associated with risk of progression from inflammatory to complicated disease.

Twenty-four of this cohort were diagnosed with UC in childhood. These children were more likely to have pan-colonic involvement and less likely to have isolated proctitis than those diagnosed over 17 years of age. Given the small size of the paediatric group, it was unclear if age at diagnosis had any impact upon disease progression over time.

Just one report has focused expressly upon IBD in children across NZ. Yap et al. [31] undertook a prospective assessment of children and adolescents diagnosed with IBD in NZ over a two-year period using an existing paediatric surveillance system to delineate incidence and presentation patterns. Fifty-two children were diagnosed over this period: 34 (66%) with CD, 9 (17%) with UC, and 9 with IBDU. The authors estimated an incidence for IBD of 2.9 per 100,000; however, this may be an underestimate given the use of a surveillance program to identify the patients.

These 52 children were diagnosed at a median age of 11 years, with a median diagnostic delay of 8.4 months. The most common symptoms seen were pain and bloody diarrhoea (63% and 57% resp.). Less than a quarter of the children diagnosed with CD presented with the triad of pain, diarrhoea, and weight loss. Forty-four (85%) of the children were described to be of European extraction, with other patients being Indian (*n* = 2), Polynesian (*n* = 3), Middle-Eastern (*n* = 2), and African (*n* = 1).

Overall, 73% of the 33 children with CD had upper gastrointestinal involvement, with colonic disease the most common location (48%). Thirteen of these children had perianal disease at diagnosis. Of the nine children with UC, eight had pan-colonic involvement, with the other child having left-sided disease.

This report was not able to provide any data on rates of paediatric IBD across NZ over a longer period of time. However, it does provide a “snap-shot” of the patterns and phenotype of disease in NZ within the two years of observation. Furthermore, this report was not designed to evaluate the wider impact of IBD in children and adolescents.

The impact of IBD upon quality of life and health care costs was evaluated in separate studies undertaken in NZ. Lowe et al. [32] evaluated quality of life (QOL) using the well-established IMPACT-III questionnaire in a small group of 16 children and adolescents with CD in the Wellington region of NZ. These children were aged between 9 and 18 years and had been diagnosed with CD for a mean of 2.7 years. Overall, the mean IMPACT-III score was 119.2 (±30.7), with maximum score of 175. Poor QOL was associated with current disease activity. Further evaluation of this group showed that many did not have peer support with other children with CD and care givers indicated a need for social supports.

The health care costs associated with CD in NZ children were evaluated in a recent study conducted in the Canterbury region of NZ [33]. Based upon a sample of 24 children diagnosed with CD, the direct and indirect health care costs were estimated to be NZ$14,375 per patient per year. Indirect costs included school absence and parental leave from work. Furthermore, an extrapolation of this data suggested an annual cost for paediatric CD of NZ$25.9 million across the country.

## 6. Discussion and Conclusions

Recent Australasian data indicate high incidence of IBD, especially CD, in both countries. Paediatric data, especially arising from Australian studies, indicates increasing rates of both CD and UC.

Prior to the large population-based studies summarised here, the patterns of IBD in Australasia were represented by a series of small reports predominantly arising from tertiary hospitals and reflecting a selection bias. Furthermore, the rates of IBD in this region were not appreciated at all, with prior studies indicating high rates of IBD in other developed countries. The recent Australasian data clearly delineate high rates of IBD, especially CD, in this region of the world.

The changing epidemiology of paediatric IBD has been described in recent reports [3, 4]. Overall, these data indicate that paediatric-onset IBD has been increasing in recent years. Reports from Ireland [34] and Scotland [35] illustrate very similar changes to those observed in Australia.

The recent Australian adult [7] and paediatric [8, 9] studies have been based in just one state of Australia (Victoria). Although it is likely that the conclusions drawn in these reports are representative of other locations in Australia, such data are not yet available. Given that Australia comprises a large land mass, with variations in climate and population distribution, it will be important to establish patterns in other locations.

The current impressions of IBD in NZ children are based upon a comprehensive population-based study in one area of NZ [6] and a national two-year prospective cohort [31]. As in Australia, it is likely that the patterns of IBD in Canterbury and Nelson reflect patterns elsewhere in NZ. However, climate and population variations along the length of NZ could influence rates in other areas. Both the Nelson and Canterbury regions of NZ have a predominantly European population. Higher numbers of Maori and Pacific Island people (for instance, in Auckland, the world's largest Polynesian city) could reflect differences in the prevalence of IBD in those areas. Though providing an impression across NZ, the paediatric surveillance study is limited by its relatively short duration and the collection method itself. Although the national paediatric surveillance program is well established, reporting relies upon the cooperation of the individual practitioners. Furthermore, some older children may have not been included if they were diagnosed by adult gastroenterologists unaware of the study.

Despite these short-comings, however, the Australasian data overall indicates high rates of IBD in children and adolescents, with increasing incidence in recent years. Furthermore, it appears that diagnosis of IBD is predominantly seen in children of European ancestry.

Generally, the available data suggest that the phenotype of IBD observed in Australasian children is similar to that observed elsewhere [36, 37]. These patterns include a predominance of CD over UC, frequent involvement of the upper gastrointestinal tract in CD, and extensive disease distribution in UC and CD.

Overall, the available data demonstrate low rates of IBD in the indigenous populations of Australia and NZ. It is not yet clear if this reflects differences in genetic risk factors or environmental factors. Variable rates of enteric infections, such as with* Helicobacter* or* Campylobacter* organisms, could be a further possible explanation [22]. Underreporting of symptoms and consequent underdiagnosis is a further possibility, but this appears less likely given the chronic and pervasive nature of IBD.

Currently there is little data on the patterns of IBD in immigrant populations in Australia and NZ. The recent studies have indicated that few individuals with IBD in NZ or Australia were first or second generation migrants [8, 9, 31]. Just one study has focused upon children from a specific ethnic group: these data demonstrated much higher rates of IBD in children whose family originated in the Middle East region [29].

Prior studies have shown that the children of individuals moving from the Indian subcontinent to countries such as Canada and the United Kingdom have higher rates of IBD upon settling in their new home compared to their region of origin or the baseline rates in their new location [38–40]. It is likely that the same patterns will be observed in Australia and NZ: specific studies focusing on these groups would be required to confirm this.

Further epidemiology studies are required in other locations throughout Australia and NZ to establish patterns of IBD across the two countries. Furthermore, natural history studies of disease cohorts in this region are required to establish and follow changes in the incidence and phenotype of disease in this region. Such studies will also help to better illustrate changes in patterns of IBD in immigrant and indigenous groups in these two countries.

## Figures and Tables

**Table 1:** Incidence of Crohn disease in Australia and New Zealand. Incidence data obtained from large population-based studies conducted in Australia and New Zealand (NZ). Incidence data represent diagnoses per 100,000 population for the relevant year.

	Canterbury, NZ	Geelong, Australia	Nelson, NZ
Year of observation	2004	2007-8	2012
Incidence	16.5	17.4	15.2
